# Chemical, Biochemical, Antimicrobial, and Pharmacological Assessment of Postdistillation Waste Material Extracts of *Mentha* x *piperita* †

**DOI:** 10.3390/ph18121782

**Published:** 2025-11-24

**Authors:** Neda Gavarić, Katarina Radovanović, Nataša Milošević, Jelena Jovičić-Bata, Mladena Lalić-Popović, Sonja Smole Možina, Isidora Samojlik

**Affiliations:** 1 Department of Pharmacy, Faculty of Medicine, University of Novi Sad, Hajduk Veljkova 3, 21000 Novi Sad, Serbia; katarina.radovanovic@mf.uns.ac.rs (K.R.); natasa.milosevic@mf.uns.ac.rs (N.M.); jelena.jovicic-bata@mf.uns.ac.rs (J.J.-B.); mladena.lalic-popovic@mf.uns.ac.rs (M.L.-P.); 2 Department of Food Science and Technology, Biotechnical Faculty, University of Ljubljana, 1000 Ljubljana, Slovenia; sonja.smole-mozina@bf.uni-lj.si; 3 Department of Pharmacology, Toxicology and Clinical Pharmacology, Faculty of Medicine, University of Novi Sad, Hajduk Veljkova 3, 21000 Novi Sad, Serbia; isidora.samojlik@mf.uns.ac.rs

**Keywords:** peppermint, deodorized extracts, antioxidant potential, hepatoprotective activity, antibacterial activity, herbal-drug interaction

## Abstract

**Background:** Aromatic plants like peppermint (*Mentha* x *piperita*, Lamiaceae) have a long tradition of use. Most of the plant material is used to produce herbal drugs and for the isolation of essential oils. However, since essential oils are present in very small amounts, the largest proportion of plants remains unused. **Objectives:** The aims of this study were the analysis of chemical, biochemical, antimicrobial, and pharmacological properties of peppermint waste material extracts (derived from stems, post-distillation waste, and deodorized leaves) in comparison with the officially prepared extract. **Results:** The obtained results revealed that the investigated peppermint waste extracts (PWEs) are a rich source of phenolic compounds, where rosmarinic acid was determined as the dominant one (7.05–21.19 mg/g d.e.). Antioxidant potential and hepatoprotective effect of PWE were comparable with the official extract, where the most active ones were those prepared by treating the deodorized leaves with both 45% and 75% ethanol. In addition, PWE exhibited notable antimicrobial and anticholinesterase activity. Results of pharmacological studies on experimental animals showed that peppermint extracts (official and those made from deodorized leaves) did not interfere with the effect of the tested drugs, midazolam and fluoxetine. The examined extracts neither exerted an influence on motor coordination nor acted as antidepressants. Results of the elevated plus maze test indicated that PWE affected the activity of the central nervous system. **Conclusions:** PWEs represent a significant source of phenolic compounds, especially rosmarinic acid, and they can be used in the pharmaceutical industry to produce various herbal products and in the food industry as natural additives.

## 1. Introduction

There are numerous recordings of peppermint (*Mentha* x *piperita* L., Lamiaceae) usage in folk medicine from ancient Egypt to modern traditional herbal medicine [1]. Hippocrates used peppermint leaves for their medicinal and digestive properties. The Romans spread it all over Europe, while the emigrants spread it to North America. Arabic medicine celebrates mint as the embodiment of friendship and love, and in many cultures, it is a symbol of hospitality (e.g., each guest is first served with mint tea). In the Middle Ages, it was used to mask unpleasant odors (Shakespeare mentioned it together with lavender and rosemary as a stimulus for medieval gentlemen), and from the 18th century, it began to be highly valued as a medicinal plant and used to treat many diseases [1]. Peppermint is native to Europe, naturalized in North America and Canada, and is cultivated in many parts of the world. The hybrid *Mentha* x *piperita* was created by crossing the species *M. aquatica* L. and *M. spicata* L. The plant is sterile, so it reproduces exclusively vegetatively by stolons. It grows on loose humus soil and requires a lot of moisture and light [2]. Peppermint belongs to the Lamiceae family, subfamily Nepetoideae, characterized by the presence of essential oil and rosmarinic acid [3]. Whole or cut, dried peppermint leaves are used as an herbal substance for preparations such as tinctures or extracts, and for the isolation of essential oil. The leaves are harvested when they are fully developed during flowering or just before the flowering of the plant [4].

The composition of secondary metabolites in peppermint leaves is very well studied, and it varies depending on the vegetation period, variety of plant, geographical region, and production conditions [5]. Peppermint leaves contain various phenolic compounds, such as flavonoids, phenolic acids, and tannins. Dominant flavonoids are luteolin and its glycoside rutin, hesperidin, eriocitrin, and highly oxygenated and methylated flavones. Rosmarinic, chlorogenic, and caffeic acids are present within the phenolic acid metabolites in the leaves. Triterpenoids, like ursolic acid, are also chemical constituents of peppermint leaves [6,7]. Peppermint essential oil consists of various mono- and sesquiterpenoids. Monoterpenoids are dominant, with menthol, menthone, isomenthone, menthyl acetate, menthofuran, and limonene 1,8-cineol as representative compounds. Also, the limits for isopulegone and pulegone in peppermint essential oil are defined in the European pharmacopeia due to their potential hepatotoxicity [8,9].

Being one of the most popular aromatic and medicinal plants globally, the usage of peppermint leaves is numerous, and the pharmacological effects are well studied. Since peppermint contains a significant amount of chemical constituents with antioxidant activity (flavonoids and phenolic acids), it is a scavenger of free radicals and inhibitor of the lipid peroxidation process [10,11]. The peppermint leaves also have a strong antimicrobial potential. The antibacterial and antifungal effect of peppermint leaves on numerous strains of bacteria and fungi is well documented [12]. This effect is primarily attributed to the essential oil [7], but other data showed that various leaf extracts have significant bactericidal and fungicidal activity too [13,14]. On the other hand, peppermint leaf extracts are also attributed to significant antiviral activity [15,16], as well as anthelmintic and insecticidal properties [17]. Peppermint leaves, as an herbal substance, and various peppermint leaf herbal preparations have been suggested to prevent or reduce carcinogenesis induced by various agents in some animal models [18]. Certain studies recorded the anti-allergic and anti-inflammatory effects of peppermint leaves, for which flavonoids and components of the essential oil are primarily responsible [15]. In addition, it has been proven that peppermint affects the activity of the central and peripheral nervous system in terms of analgesic and local anesthetic effects attributed to the action of menthol [19,20]. Recent studies have shown that peppermint ethanolic extracts exhibit anticholinesterase activity and prevent neurodegeneration in animal models and partially maintain dopamine levels [21]. Well-established usage of peppermint leaves and peppermint essential oil includes symptomatic relief of minor spasms of the gastrointestinal tract, flatulence, and abdominal pain, especially in patients with irritable bowel syndrome. Also, cutaneous application of peppermint essential oil is used for the symptomatic relief of mild tension-type headaches [4]. Traditional usage of peppermint leaves and their preparations also includes the relief of symptoms in coughs and colds, the symptomatic relief of localized muscle pain, and pruritic conditions in intact skin [4]. The usage of peppermint leaves and their preparations in therapeutic doses in the abovementioned indications is safe considering all contraindications, special warnings, and precautions for use [4]. In addition, both peppermint leaves and peppermint essential oil are generally recognized as safe (GRAS) for specific uses [22]. Regarding the wide use of various peppermint preparations and their massive production, it is important to enhance the exploitation of the produced plant material for both ecological and economic reasons. Hence, the aim of this study was to evaluate chemical, biochemical, antimicrobial, and pharmacological properties of post-distillation PWEs.

## 2. Results and Discussion

### 2.1. Extraction Yield and Chemical Composition

The amount of dry extract gained from untreated leaves (P1) (Table 1) generally corresponds to the data reported in the literature [10,23]. However, it should be borne in mind that the extraction methodology rarely fully matches the various literature references. Many factors, such as the polarity of the extractant, grinding of the plant material, the drug-to-solvent ratio, the temperature and pressure in the system, and the method of filtration, affect the yield of the dry extract [24]. Also, various exogenous factors such as the degree of insolation, type, humidity, and composition of the soil can affect the content of dry extract in the plant material. As for the extracts obtained by treating the peppermint post-distillation waste material, the largest amount of dry extract was gained by evaporation of the post-distillation residue (P2) (24.17%) (Table 1), which is in accordance with previously published data [25,26,27].

The significant difference in dry extract content between the standard P1 extract and P2 can be partially explained by the production process itself. Namely, by boiling the drug in water (hydro-distillation process), larger amounts of polar substances are extracted, not only various phenolics but also free sugars present in the plant material or formed by the hydrolysis of glycosides. The amount of dry extract obtained by cold maceration of the deodorized peppermint leaves (P3, P4) is smaller compared to the amount obtained from the untreated leaf (P1). The amount of dry residue gained by treating the ground peppermint stems is also smaller compared to the P1, with 45% ethanol as a preferred extractant (Table 1).

The concentration of total phenolic compounds of PWE varies from 24.14 (P5) to 38.73 (P4) mg GAE/g d.e., while the content of total flavonoids ranges from 10.44 (P5) to 67.63 (P4) mg GAE/g d.e. The P2 extract contains a slightly lower concentration of total phenols compared to literature data [26,28]. Regarding the content of total phenols and flavonoids, extract P4, obtained by treating the deodorized peppermint leaves with 75% ethanol, stands out, especially in terms of total flavonoids, where the highest value was obtained in relation to all analyzed extracts. This result can be partially explained by the dominance of less polar, methylated flavonoid glycosides in the peppermint leaves, which hydrolyze into aglycone and sugar components when boiled in water. Released aglycones are well extracted with 75% ethanol as a less polar extractant [6]. The peppermint stem extracts P5 and P6 contain a slightly lower amount of total phenolics and flavonoids compared to the other investigated PWE, while the higher content was obtained using 75% ethanol as an extractant (P6).

**Figure 1:**
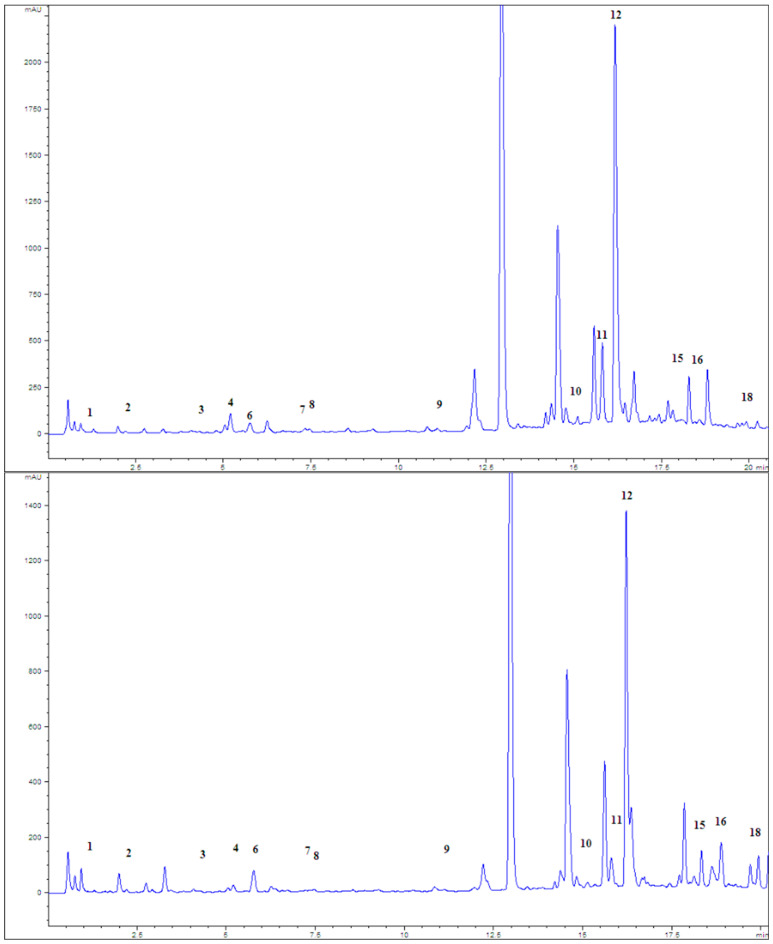
Chromatograms of examined peppermint extracts P1 (above) and P4 (below), at 280 nm; 1—gallic acid, 2—protocatechuic acid, 3—catechin, 4—caffeic acid, 6—chlorogenic acid, 7—syringic acid, 8—epicatechin, 9—ferulic acid, 10—rutin, 11—myricetin, 12—rosmarinic acid, 15—naringenin, 16—luteolin, and 18—apigenin.

The following phenolic compounds were quantified by chromatographic analysis of PWE: gallic, protocatechuic, caffeic, chlorogenic, syringic, ferulic, and rosmarinic acids, as well as catechin, epicatechin, apigenin, naringenin, and rutin [29]. The results are presented in Table 2, and the corresponding chromatograms are given in Figure 1.

Within the fraction of phenolic acids, rosmarinic acid was identified as the dominant compound in all tested peppermint extracts. Its concentration varies from 7.05 (P5) to 21.19 (P4) mg/g d.e., which is in accordance with previously published data for the extract P1 [12,30]. The results for tested phenolic acids in the decoction remaining after the isolation of the essential oil (P2) align with the available literature [26,27].

Within the flavonoid fraction, apigenin is present in the highest concentration (from 0.31 (P5) to 3.07 (P3) mg/g d.e.) [31]. Interestingly, the concentration of apigenin increased in deodorized extracts compared to the untreated peppermint leaf extract. This may be partially explained by the thermal and enzymatic release of apigenin from glycosides and improved extractability due to tissue disruption [6,32]. This phenomenon has also been reported in other flavone-containing plants [31].

The extracts prepared by treating the deodorized peppermint leaves (P3 and P4) stand out for the content of quantified phenolic compounds, especially P4. These findings indicate that the analyzed post-distillation waste extracts represent a good source of phenolic compounds, with rosmarinic acid in first place.

### 2.2. Antioxidant Potential

The assessment of antioxidant activity of a natural product begins with *in vitro* tests, which are cheaper and more ethically acceptable than *in vivo* models. However, regarding the complex nature of the oxidative processes in organisms, it is recommended to perform several tests with different mechanisms of antioxidant protection to assess this potential more objectively [33]. Therefore, we examined the RSC of PWEs on DPPH and OH radicals, and their influence on the lipid peroxidation process.

#### 2.2.1. Antioxidant Activity *In Vitro*

Table 3 shows the IC_50_ values of the investigated PWE in DPPH, OH, and LP tests. All tested peppermint extracts neutralize the DPPH radical in a concentration-dependent manner, with the percentage of neutralization ranging from 4.95 to 85.08 at concentrations of extracts from 1.25 to 12.5 µg/mL. Moreover, all tested extracts, except P5, where the lowest concentration of phenolic compounds was determined (Table 1), exhibited a very strong “scavenger” activity on DPPH, which corresponds to the available literature data [7,12,14]. Examined extracts exhibited stronger activity compared to BHT. This may indicate their potential application as antioxidants in the food and pharmaceutical industry. On the other hand, isolated rosmarinic acid and rutin exhibited stronger antioxidant activity compared to the examined peppermint extracts, which is in accordance with data from the literature [12]. This may indicate the eventual application of the tested post-distillation waste for the isolation of these and similar phenolic compounds. Although the DPPH radical is a synthetic one, the results of this test can be used as a preliminary indicator of antiradical activity and can be used for defining antioxidant activity in combination with the results obtained in other tests.

To obtain a more complete picture of the antiradical potential of the examined extracts, their effect on the hydroxyl radical (OH^•^), the most chemically reactive reduced form of oxygen, which is therefore the most responsible for its toxic effects, was also determined [34].

Among the examined peppermint extracts, the extract of the deodorized leaves treated with 45% ethanol (P3) stands out for its ability to neutralize OH^•^, although P4 is characterized as the richest in rosmarinic acid (Table 3). Such a prominent action of the P3 extract can be partially explained by the optimal ratio of phenolic compounds, favorable for the neutralization of OH^•^. On the other hand, P4 was the extract with the highest concentration of total flavonoids. However, peppermint leaves are characterized by the presence of methylated flavonoid aglycones [6], which have a reduced number of OH groups available for participation in free radical reactions [35]. The percentage of neutralization of OH^•^ by the tested peppermint extracts ranged from 13.97 to 73.43 at concentrations of extracts 8.33 to 166.7 µg/mL, which is in accordance with the literature [36].

Investigated PWE inhibited the process of peroxidation of unsaturated fatty acids in corn oil. The percentage of LP inhibition ranged from 8.86 to 85.2 when concentrations of extracts 2.5–50 µg/mL were applied. Almost all tested extracts showed a strong inhibitory effect on LP, and stronger when compared to isolated rosmarinic acid (Table 3). This result indicates a unique synergism of phenolic compounds present in the tested extracts on the complex process of lipid peroxidation [37]. Additionally, all tested peppermint extracts showed a stronger effect on LP when compared to BHT, which contributes to their possible use as antioxidants of natural origin in food and pharmaceutical industries. Natural antioxidants could be considered both ecological and non-toxic, but also effective enough to prevent oxidative damage of various products.

#### 2.2.2. Antioxidant Activity *Ex Vivo*

Hepatoprotective activity of investigated peppermint extracts on mice liver was assessed by detecting its influence on lipid peroxidation, reduced glutathione concentration, and activity of enzymes responsible for oxidative processes (XOD, SOD, GSH-Px, GSH-R, and GSH-(S)T). P3 and P4, made from deodorized peppermint leaves using 45% or 75% ethanol, were singled out for these experiments due to their chemical composition and strong antioxidant potential in *in vitro* tests.

The most notable results were obtained for the influence on LP and GSH concentration. Assessing the effect on the LP process, it was found that all tested extracts led to a statistically significant decrease in the intensity of lipid peroxidation in the liver homogenate of experimental animals compared to the group treated with CCl_4_, while the P4 extract led to a statistically significant decrease in LP compared to the control (Table 4).

CCl_4_, as a hepatotoxic agent, increases the activity of enzymes of primary antioxidant defense, thereby consuming reduced glutathione. Extracts P3 and P4 prevent a significant drop in GSH concentration after CCl_4_ administration. Namely, the concentration of GSH is significantly higher in the groups of experimental animals pretreated with peppermint extracts when compared to the group that received CCl_4_ only.

In addition, P4 leads to a statistically significant increase in GSH concentration compared to the control group. These results indicate the antioxidant potential of the waste material that remains after the isolation of peppermint essential oil. Similar results were obtained in the study of peppermint ethanolic extract influence on LP and GSH in the mouse brain homogenate [21].

When monitoring the effect of the investigated extracts on the activity of enzymes responsible for oxidative processes (XOD, SOD, GSH-Px, GSH-R, and GSH-(S)T), it was noted that both untreated and PWE do not show a protective effect in terms of reducing the activity of the mentioned enzymes as an oxidative stress response. Yet, examined extracts do not exhibit a statistically significant effect on their activity compared to the control group. This may indicate safe application of waste extracts and confirm their GRAS status.

### 2.3. Antibacterial Testing

Numerous studies have confirmed the use of medicinal plants as anti-infective agents. Among them, various phenolic compounds or plant extracts rich in these chemical constituents stand out for their antimicrobial potential [38]. This is particularly important since phenolic compounds also exhibit a strong antioxidant potential, so this combined action is significant not only in the pharmaceutical and food industry, but also in all other areas where it is important to prevent oxidation and contamination of a certain product [25]. Since the investigated PWEs possess antioxidant potential, the antimicrobial action was determined on several bacterial strains, such as *Staphylococcus aureus*, *Salmonella* Infantis, *Escherichia coli*, and *Bacillus cereus* (Table 5). Analyzed peppermint extracts showed a relatively strong antimicrobial potential against all tested strains, with P3, the extract obtained from the deodorized leaves using 45% ethanol, being the most effective. Comparing these results with the data obtained for BHT, it is significant to emphasize that all peppermint waste extracts are more potent antibacterial agents. Also, extracts exhibit stronger antibacterial activity than isolated rosmarinic acid, indicating possible synergism against tested bacterial strains [12,14].

To test bacteriostatic activity on *Campylobacter jejuni,* P3 was chosen and compared with P1 (Table 6). This bacterial strain represents a particular problem in meat production and processing, often being the cause of food poisoning. The selected extracts showed a relatively strong antibacterial effect on *Campylobacter jejuni*, where P3 was more potent than P1, an officially prepared peppermint leaf extract. Also, examined extracts displayed a stronger anti-campylobacter activity than isolated rosmarinic acid, but were less potent than the tested antibiotics.

The results of microbiological tests indicate the possibility of using PWE primarily in the food industry as natural additives and for the isolation of the dominant compounds, phenolic acids and flavonoids, most responsible for the pronounced antimicrobial action.

### 2.4. Anticholinesterase Activity

**Figure 2:**
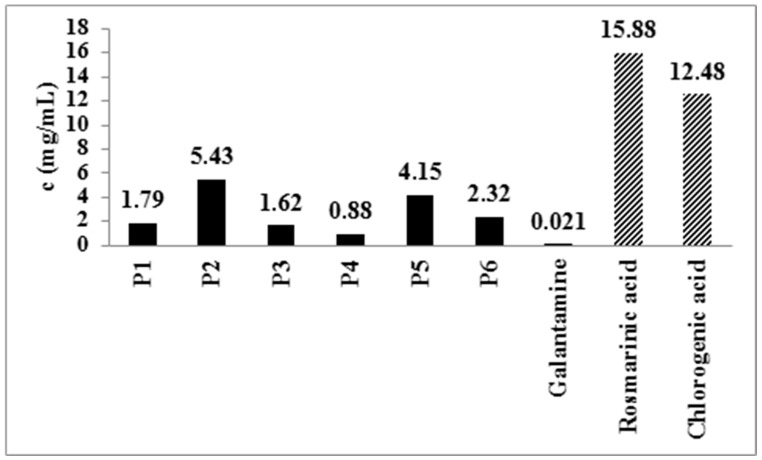
IC_50_ values (mg/mL) of the inhibitory effect of the examined peppermint post-distillation waste extracts, galantamine, rosmarinic, and chlorogenic acid on the acetylcholinesterase enzyme.

Various medicinal plants can be useful in the prevention and treatment of central nervous system diseases [21]. The main mechanisms of action that can alleviate symptoms of Alzheimer’s disease (AD) include the inhibition of AChE, which is often associated with pronounced anti-inflammatory and antioxidant activity exhibited by phenolic compounds such are flavonoids and phenolic acids [39]. Considering the abovementioned facts, the activity of peppermint extracts, both the standard extract (P1) and PWEs (P2-P6), on the enzyme AChE was monitored. Galantamine was used as a positive control, and the effect of isolated rosmarinic acid was also investigated. The tested peppermint extracts exhibited an inhibitory effect on AChE, with IC_50_ values ranging from 0.88 (P4) to 5.43 (P2) mg/mL (Figure 2), which is consistent with formerly presented data [40]. These concentrations for examined peppermint extracts are higher than previously published, while the IC_50_ values for galantamine are similar. However, the above-mentioned study examined solely non-polar peppermint extracts [41]. Extracts of the deodorized leaves showed the strongest effect, especially P4, which is the most abundant source of quantified phenolic acids and flavonoids (Table 2). In addition to the inhibitory effect on AChE, extracts rich in flavonoids can have a therapeutic potential in AD due to the ability of flavonoids to protect the brain from the action of neurotoxins and various inflammatory processes, to activate synaptic transmission, and improve circulation in the brain [21].

Also, all tested peppermint extracts showed a stronger effect when compared to isolated phenolic acids, which indicates a synergistic action of phenolic compounds in AChE inhibition.

### 2.5. Pharmacodynamic Studies

It is of utmost importance to thoroughly investigate the pharmacological activity of herbal medicinal products by performing various *in vivo* tests, but also to elucidate possible interactions with conventional medicines [42]. Pharmacodynamic studies of selected peppermint extracts, both untreated (P1) and deodorized leaf extracts (P3 and P4), included the following: their influence on motor coordination, antidepressant and anxiolytic effects, as well as the effect on the memory of experimental animals. In addition, the possibility of interactions between the examined extracts and drugs from the group of sedatives (midazolam) and antidepressants (fluoxetine) was tested.

#### 2.5.1. Rotarod Performance Test

The rotarod performance test is a simple pharmacodynamic test for monitoring the influence of synthetic drugs, as well as natural products (isolated compounds or various extracts) on the motor coordination of experimental animals. Benzodiazepine midazolam, a hypnotic with an effect on muscle tone and coordination, was used as a positive control. It is characterized by a fast onset and a short action. It is metabolized mainly in the liver via the CYP3A4 isoenzyme, especially if it is applied parenterally [19]. In addition to being used for its effect on motor coordination, its intraperitoneal application for experimental purposes is also suitable for indirect testing of potential interactions at the level of the liver enzyme.

Midazolam, administered intraperitoneally at a dose of 5 mg/kg, led to a statistically significant disturbance of motor coordination in all tested groups of animals. It was manifested as a shortening of the time that animals spent on the rotating rod (especially in the first and fifth min. of the testing period after drug application) compared to the control group of animals, the ones that did not receive the drug. None of the tested peppermint extracts showed any effect on the motor coordination of the experimental animals after repeated oral administration of the doses shown in Section 3.4.2. (Table 7). During the simultaneous application of the investigated peppermint extracts (P1, P3, and P4) and midazolam, it was determined that the extracts do not have a statistically significant effect on the pharmacodynamics of the applied drug. In these groups of experimental animals, there was neither an extension nor a shortening of the time spent on the rotating rod, compared to the group that received only midazolam.

#### 2.5.2. Tail Suspension Test

The Tail Suspension Test is one of the most applied pharmacodynamic tests for evaluating the antidepressant effect of synthetic drugs, as well as natural products. The test induces a state of behavioral despair in experimental animals, characterized by a period of immobility after initial escape-oriented movements. The reduction in immobility time is considered to reflect the antidepressant effect of the tested compounds. Fluoxetine, which was used as a positive control, is an antidepressant from the group of selective serotonin reuptake inhibitors (SSRIs) with a long half-life (24–96 h), which increases the possibility of interactions with other synthetic and herbal drugs [43]. Fluoxetine led to a statistically significant shortening of the period of immobility in all tested groups of animals, compared to the control group (Table 8). On the other hand, none of the examined extracts showed an antidepressant effect. Immobilization times in these groups of animals were very close to control values.

In a recent study, aqueous peppermint extract exhibited a reduction in immobility time in TST after 28 consecutive days of treatment [44]. These findings indicate that peppermint extracts should be applied for a longer period to assess antidepressant activity.

The concomitant application of examined peppermint extracts and fluoxetine showed no pharmacodynamic interaction, i.e., no statistically significant differences in immobilization times were determined. Based on these results, it can be concluded that the tested peppermint extracts do not significantly change the mechanism of action of the drug and do not affect the turnover of serotonin. This data is significant because it indicates that there is no risk of various side effects (e.g., serotonergic syndrome) during concurrent use of fluoxetine and peppermint extracts.

#### 2.5.3. Elevated Plus Maze

**Figure 3:**
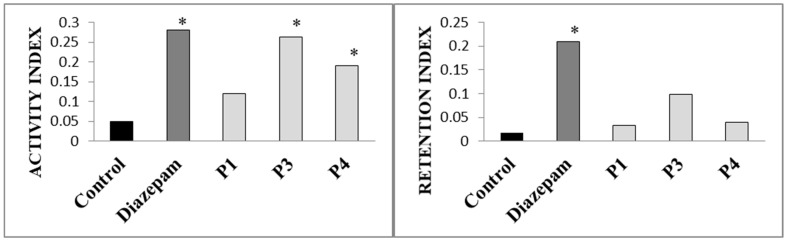
Activity index and retention index in the EPM after application of diazepam and tested peppermint extracts; *—*t*-test confirmed the statistically significant difference compared to the control (*p* < 0.05).

One of the tests used to evaluate the anxiolytic action of various compounds of natural or synthetic origin is the elevated plus maze test. Diazepam, a benzodiazepine anxiolytic, is used as a positive control [45]. Diazepam showed an anxiolytic effect that was manifested by a statistically significant increase in the activity and retention index compared to the control group (Figure 3). Namely, diazepam caused an increase in the number of entries into the open arms of the elevated plus maze and increased the time that animals spent in the open arms (Table 9).

Analyzed peppermint extracts led to a statistically significant increase in the activity index compared to the control group of animals. However, although P3 caused a statistically significant increase in the time spent in the open arms (Table 9), neither P3 nor P4 produced a significant increase in the retention index (Figure 2). This result indicates the influence of peppermint leaf extracts on the increased activity of the central nervous system, possibly through the dopaminergic system, with the absence of an anxiolytic effect [21].

Various studies discussed the relevance of phenolics to the dopaminergic system and the possible use of phenolic-rich extracts in dopaminergic disorders. Namely, it is confirmed that peppermint ethanolic extracts elevate the expression of genes related to dopamine, suggesting the modulation of synapses and maintenance of motor function in the rotenone-exposed mouse model [21]. Also, an elevated expression of antioxidant marker genes was registered, indicating the possible augmented biogenesis of mitochondria in mid-brain neurons. Moreover, the neuroprotective effects of various flavonoids, specifically flavones identified in peppermint leaves, have been well documented [46]. These therapeutic properties are achieved through protection of ACH receptors from reactive oxygen species, impact on AChE expression, AChE and MAO inhibition, action on GABA-A receptors, and effects on glutamate, 5-hydroxytryptamine, and dopamine signaling [46].

#### 2.5.4. Novel Object Recognition Test

Neurodegenerative diseases represent one of the biggest problems facing the healthcare system today. One of the symptoms of some of those diseases is episodic memory loss. NOR is used in the search for new, effective synthetic or natural drugs that can influence the memory in experimental animals [43].

The tested peppermint extracts, prepared from both untreated leaves (P1) and the waste material remaining after the isolation of the essential oil (P3 and P4), did not show a significant effect on the memory of experimental animals in this test.

Namely, after repeated oral application of the peppermint extracts in the doses given in Section 3.4.2, there was no statistically significant increase in the d2 index, as a measure of discrimination between known and new objects. The highest value of d2 (0.42) was achieved after the application of extract P4. Perhaps a more significant difference between the group of animals receiving the extract and the control group would be obtained if the dosing regimen were changed, in terms of increasing the dose of P4 extract and extending the time of its application. There is no data dealing with the effect of peppermint extract in NOR.

## 3. Materials and Methods

### 3.1. Plant Material, Extract Preparation (P1–P6) and Extraction Yield

The above-ground parts of cultivated peppermint (*Mentha* x *piperita* L., Lamiaceae) were collected in Padej, Vojvodina Province, Republic of Serbia, just before flowering. The leaves were separated from the stems, and the plant material was air-dried in a shaded, well-aerated location until constant mass was obtained. Dried plant material was deposited in paper bags in a dry and cool place for further analysis at the Herbarium of the Laboratory of Pharmacognosy, Department of Pharmacy, Faculty of Medicine, University of Novi Sad, Serbia.

Officially prepared peppermint leaf extract (P1) was macerated in 45% ethanol (EtOH) for 24 h at 23 °C (drug-to-solvent ratio was 1:10, *w*/*v*) (Table 10) [4]. The hydro-distillation technique was applied for the isolation of essential oil [8]. The waste plant material used for further processing was filtered (pore size 40 µm), and the remaining water extract (decoction) was extract P2. Deodorized leaves were air-dried and macerated with 45% and 75% EtOH for 24 h at 23 °C to produce extracts P3 and P4, respectively. Ground dried stems (sieve 0.75) were macerated with 45% and 75% EtOH, 24 h at 23 °C to obtain stem extracts P5 and P6, respectively. After the maceration, extracts were collected and evaporated to dryness under a vacuum (Laborota 4001 efficient, Heidolph, Schwabach, Germany).

The extraction yield was calculated according to the following equation:

Extraction yield (%) = m_de_/m_pm_ × 100 (1)

where m_de_ is the mass of dry extract and m_pm_ is the mass of plant material used for extraction.

Residues were dissolved in water to make 10% (*w*/*v*) stock solutions for chemical, biochemical, microbiological, and pharmacological analysis, as well as in a methanol:1% formic acid mixture (50:50 *v*/*v*) to make 2% (*w*/*v*) stock solutions for the HPLC analysis.

### 3.2. Experimental Animals

Six-week-old male Swiss Albino mice, weighing 25–35 g, bred at the experimental animal farm of the Military Medical Academy in Belgrade, were used in the tests. Animals were housed in their individual cages 7 days prior to pharmacodynamics and *ex vivo* antioxidant studies under controlled conditions (temperature 23 ± 1 °C, constant air humidity of 60% and 12 h day-night cycle), and they were provided with food and water *ad libitum*. All efforts were made to minimize animal discomfort, and the experimental procedures were approved by the Ethical Committee for Animal Use in Experiments, University of Novi Sad. The experiments were conducted at the Department of Pharmacology, Toxicology and Clinical Pharmacology, Faculty of Medicine, University of Novi Sad, Serbia.

### 3.3. Chemical Composition

A previously described spectrophotometric method using Folin–Ciocalteu reagent was utilized to determine the total phenolics (TPs) [47]. The content of TP was expressed in milligrams of gallic acid equivalents per gram of dry extract (mg GAE/g d.e.).

**Figure 4:**
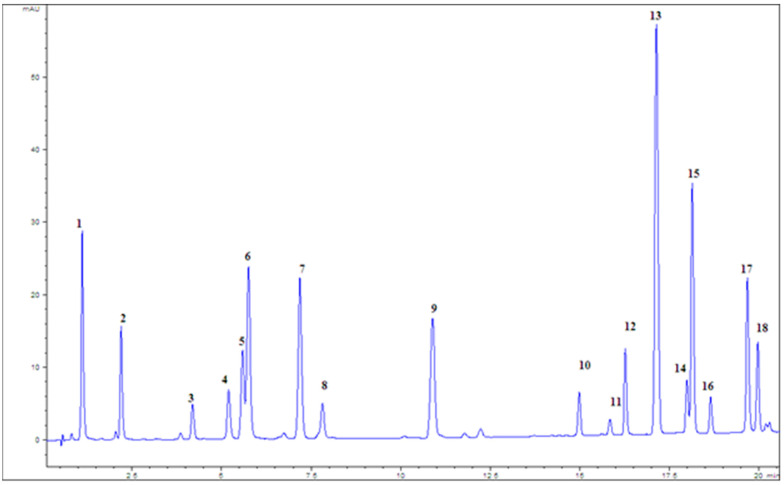
Chromatogram of the mixture of standards at 280 nm; 1—gallic acid, 2—protocatechuic acid, 3—catechin, 4—caffeic acid, 5—vanillic acid, 6—chlorogenic acid, 7—syringic acid, 8—epicatechin, 9—ferulic acid, 10—rutin, 11—myricetin, 12—rosmarinic acid, 13—cinnamic acid, 14—quecetin, 15—naringenin, 16—luteolin, 17—kaemferol, and 18—apigenin.

The total flavonoid (TF) content was determined based on the flavonoid–AlCl_3_ reagent complex formation and showing the maximum absorption at 430 nm. The results were expressed as milligrams of quercetin equivalents per gram of dry extract (mg QE/g d.e.) [47]. Detailed chemical analysis, in order to quantify selected phenolic compounds, was performed by using a liquid chromatograph (Agilent 1200 series), equipped with a diode array detector (DAD) (both from Agilent Technologies, Waldbronn, Germany) and Eclipse XDB-C18, 1.8 μm, 4.6 × 50 mm column, at a flow rate of 1 mL/min. A solvent gradient was performed by varying the proportion of solvent A (methanol) to solvent B (1% formic acid in water (*v*/*v*)) [48]. The total running time and post-running time were 45 and 10 min, respectively. The column temperature was 30 °C. The injected volume of samples and standards was 5 μL, and it was performed automatically using an autosampler. The spectra were acquired in the range of 210–400 nm, and chromatograms were plotted at 280, 330, and 350 nm with a bandwidth of 4 nm, and with reference wavelength/bandwidth of 500/100 nm. Quantification of selected phenolic compounds was performed by using the calibration curve of standard compounds (Figure 4).

### 3.4. Antioxidant Potential

The antioxidant activity of the examined peppermint post-distillation waste extracts (PWE) was assessed through a series of *in vitro* tests followed by *ex vivo* experiments.

#### 3.4.1. *In Vitro* Antioxidant Activity

The investigation of *in vitro* antioxidant activity was performed by measuring the free radical scavenging capacity (RSC) of PWEs on DPPH and OH radicals. These assays were combined with the determination of influence on the lipid peroxidation (LP) process. All measurements were performed in triplicate with a series of extract concentrations. The percent of RSC or inhibition of lipid peroxidation was calculated by the following equation:

RSC/I (%) =100% × (A_control_ − A_sample_)/A_control_ (2)

IC_50_ values, which represent the concentration of the examined extract causing 50% of free radicals’ neutralization or lipid peroxidation inhibition, were calculated from RSC/I (%) values by regression analysis.

DPPH assay was performed by a previously described method [49] where the neutralization of DPPH radicals by the examined extracts was measured spectrophotometrically at 515 nm. Since the final concentration of DPPH radical influences the IC_50_ value, we calculated the antioxidant activity index (AAI) to standardize the results and make them comparable with other studies. AAI was calculated as follows:

AAI = DPPH concentration in reaction mixture (µg/mL)/IC_50_ (µg/mL). (3)

Investigated extracts and isolated chemical constituents or synthetic antioxidants were classified as showing poor, with AAI < 0.5; moderate, with 0.5 < AAI < 1; strong, with 1 < AAI < 2; and very strong antioxidant activity, with AAI > 2 [50]. Isolated rosmarinic acid, rutin, and tert-butylhydroxytoluene (BHT) were also tested for comparison.

The neutralization of the OH radical, generated in the Fenton reaction, by the examined peppermint extracts was evaluated by measuring the 2-deoxy-D-ribose degradation with OH radicals [51].

Inhibition of lipid peroxidation was determined in the Fe^2+^/H_2_O_2_ system of induction spectrophotometrically. The method was described in detail previously [47]. The extent of LP was assessed by measuring the color of adduct produced in the reaction of tiobarbituric acid with malonyldialdehyde (MDA), the final oxidation product in the peroxidation of lipids [52]. Rosmarinic acid and BHT were also tested for LP inhibition.

#### 3.4.2. *Ex Vivo* Antioxidant Activity

After the analysis of chemical constituents and *in vitro* antioxidant potential, P3 and P4 extracts were singled out for the determination of *ex vivo* antioxidant (hepatoprotective) activity. The P1 extract was used for comparison. Experiments were conducted on mice liver homogenate. All experimental animals were divided into three principal groups (*n* = 10) according to the oral treatment, receiving a daily dose of P1, P3, and P4, during 5 consecutive days. The control group of animals received only water. Human daily doses of examined peppermint extracts recommended by the EMA monograph were adapted for experimentation on mice by the conversion equation for the human equivalent dose (HED) proposed in Guidance for Industry and Reviewers [53], as follows:

HED (mg/kg) = animal dose (mg/kg) × (animal weight (kg)/human weight (kg))^0.33^. (4)

**Figure 5:**
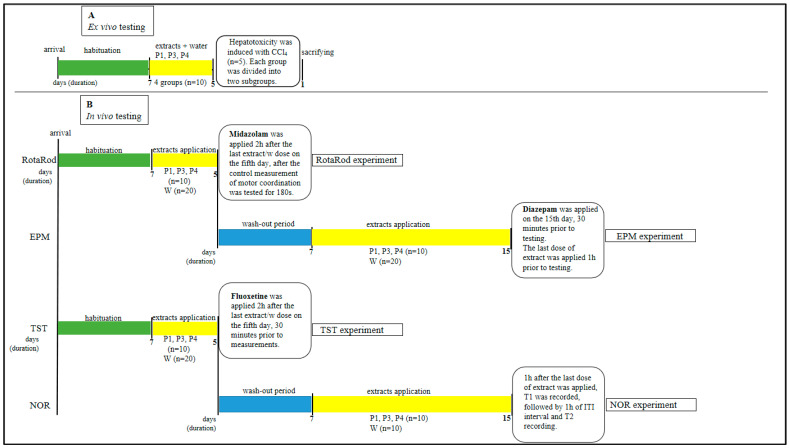
Timeline of experimental plan of *ex vivo* and *in vivo* experiments. P1—standard extract; P3—deodorized leaf extract (45% ethanol); P4—deodorized leaf extract (75% ethanol); W—water; EPM—Elevated Plus Maze; TST—Tail Suspension Test; and NOR—Novel Object Recognition.

The dose for P1 was calculated at 1.67 g/kg, and it was also used for P3 and P4. After 5 days of consecutive treatment with the examined extracts, each group of animals was divided into two subgroups (*n* = 5), including the control group (Figure 5). Hepatotoxicity induction with CCl_4,_ together with the homogenization process, was described in detail in [47].

The influence of examined peppermint extracts on the activity of liver enzymes was determined spectrophotometrically. The Agilent 8453 UV/VIS (Agilent Technologies, Waldbronn, Germany) was used. The following parameters were measured:

1. Total protein content;
2. Activity of xanthine oxidase (XOD);
3. Activity of superoxide dismutase (SOD);
4. Lipid peroxidation (LP);
5. Glutathione cycle, determined by
  - Total glutathione content (GSH);
  - Activity of glutathione peroxidase (GSH-Px);
  - Activity of glutathione reductase (GSH-R);
  - Activity of glutathione-S-transpherase (GSH-(S)T) [47].

### 3.5. Antibacterial Testing

#### 3.5.1. Bacterial Strains and Growth Conditions

The following bacterial strains from the Food Microbiology Laboratory, Department of Food Science and Technology, Biotechnical Faculty, University of Ljubljana were used to determine the antimicrobial activity of the tested extracts: *Staphylococcus aureus*, *Bacillus cereus*, *Salmonella* Infantis, *Escherichia coli*, and *Campylobacter jejuni* (Table 11).

Tested cultures, except for *Campylobacter jejuni*, were stored in Brain Heart Infusion (BHI) liquid medium with the addition of glycerol (850 µL culture in BHI and 150 µL glycerol) in a cryotube at −20 °C. The revitalization of the strains was performed by adding the contents of the cryotube to 4 mL of BHI, followed by incubation for 24 h at 37 °C with stirring (100 rpm). After incubation, the cultures were sown on selective media (24 h, 37 °C) for identification, and then on nutrient medium Mueller Hinton agar (MHA) (24 h, 37 °C), where they were stored in aluminum foil at −8 °C until the start of the experiment [54]. The *Campylobacter jejuni* strain was stored in a cryotube at a temperature of −80 °C. Revitalization of the strain was carried out by sowing on plates with Columbia blood agar, which were then incubated for 24 h, under microaerophilic conditions, at 42 °C [55].

#### 3.5.2. Broth Microdilution Method

The broth microdilution method was used to elucidate the antimicrobial potential of the examined PWE. Bacterial growth was assessed visually using INT (2-[4-iodophenyl]-3-[4-dinitrophenyl]-5-phenyltetrazolium chloride). The change in color occurs due to the reduction process, as INT acts as an artificial terminal electron acceptor in respiration. Nevertheless, tetrazolium salts are not appropriate for microaerophilic campylobacters since they indicate respiratory activity. Therefore, we used the broth microdilution method with ATP measurement as a rapid and accurate tool for anti-campylobacter activity [56]. Due to the specific conditions for the investigation of antimicrobial activity on *Campylobacter jejuni*, P1 extract, as the officially prepared one, and P3 extract, as a PWE with a high concentration of phenolics with notable antioxidant activity, were singled out for this method. Both procedures were described in detail in [54]. The wells with a bacterial suspension in an appropriate growth medium and a bacterial suspension in an appropriate growth medium with ethanol in amounts corresponding to the highest quantity present in the broth microdilution assay were used as a positive control. Negative controls were wells with growth medium and plant extract. All measurements of MIC values were repeated in triplicate. Results are expressed in mg of total phenolics (TPs).

### 3.6. Anticholinesterase Activity

Anticholinesterase potential of the peppermint post-distillation waste extracts was estimated by modified Ellman’s method [57]. The rate of yellow (Ellman’s) anion formation by the activity of acetylcholinesterase (AChE) was measured continuously spectrophotometrically at 410 nm. The final activity of the enzyme in the reaction mixture was 8.15 U/L. The degree of inhibition of AChE (% I) by the tested samples was determined based on the following formula:

$$\%I=100-\;\frac{A_{sample}\;\times 100}{A_{control}}$$

The values of 50% inhibition of the AChE (IC_50_ values) were determined by regression analysis of values obtained for the inhibitory action of a series of increasing concentrations of examined extracts and isolated rosmarinic and chlorogenic acids. Galantamine was used as a positive control. All measurements were performed in triplicate.

### 3.7. Pharmacodynamic Studies

Pharmacodynamic studies were performed on three experimental groups receiving P1, P3, and P4 extract (ten animals in each group), on a group of animals that received neither the drug nor the extract (negative control), as well as on a group of animals that received only the tested drug (positive control). Pharmacodynamic studies included testing the effect of the examined peppermint extracts on motor coordination, antidepressant and anxiolytic effects, and the effect on the memory of the experimental animals. Additionally, the possible herbal–drug interactions of tested extracts with midazolam, fluoxetine, and diazepam were assessed [43]. The timeline of *ex vivo* and *in vivo* experiments is presented in Figure 5.

### 3.8. Rotarod Performance Test

The rotarod performance test was conducted as described in [19]. Midazolam solution (5 mg/kg) was applied intraperitoneally 2 h after the last oral intake of the examined peppermint extracts, on the fifth day of treatment. The control measurement of motor coordination (assumed 180 s) was performed before the drug application. The depressive-hypnotic effect of the tested extracts and midazolam was calculated as the percentage of maximum time spent on the rotarod:

% depression= ((A × 100)/B) − 100

where A is the measured time (s) and B is the control time (180 s).

#### 3.8.1. Tail Suspension Test (TST)

The potential antidepressive effect, as well as possible interaction of the examined peppermint extracts with fluoxetine, were assessed using TST. The apparatus was described in detail in [43]. The tested extracts were administered orally, for five consecutive days, after which the immobilization time (IT) was measured [19,58]. Fluoxetine (32 mg/kg) was administered intraperitoneally on the fifth day, 2 h after the last oral dose of the extract, and the IT was measured after 30 min. The IT was recorded for a 6 min. period. The experimental animals that participated in the study were transported to the room where the measurement was performed at least one hour before the test to exclude the influence of relocation on the test results.

#### 3.8.2. Elevated Plus Maze Test (EPM)

To determine the anxiolytic effect of peppermint standard (P1) and deodorized leaf extracts (P3 and P4), the EPM test was used. The maze and experimental details were described in [43]. To rule out the effect of reentering the maze on the results, experimental animals entered the EPM only once during the experiment [45]. The anxiolytic effect of the examined peppermint extracts was assessed with two parameters: activity index or ratio entries, which represents the number of experimental animals entering the open arms of the maze divided by the total number of entrances, and retention index or time ratio, which represents the time spent in open arms divided by time spent in all arms during measurements [59]. The increased anxiety in experimental animals was reflected in low values of monitored indices. The tested extracts were administered orally for 15 consecutive days, with the last dose 1 h prior to the EPM test. Diazepam was used as a positive control at a dose of 1.5 mg/kg, administered intraperitoneally 30 min before the EPM test [59].

#### 3.8.3. Novel Object Recognition Test (NOR)

The influence of the examined peppermint extracts on the episodic memory of experimental animals was monitored using NOR. The experimental design was described in detail in [43]. The whole procedure was divided into 3 time segments: trial 1 (T1), intertrial interval (ITI, 1 h), and trial 2 (T2). The following parameters were recorded: the time spent next to the object (s) in T1 (this is the first contact with objects a1 and a2) and the time spent next to the object in T2 (where one object from T1 is replaced with a novel object—b). Subsequently, the following parameters were calculated based on the recorded times:

e1 = a1 + a2—as time spent next to identical objects in T1;

e2 = a + b—cumulative time spent next to the old and new—replaced object in T2;

d1 = b − a—absolute difference;

d2 = d1/e2—relative difference, as a measure of discrimination between a known and a new object (discrimination index).

The extracts were administered orally for 15 consecutive days, with the last dose 1 h before T1. NOR was also performed with a control group of animals (which did not receive the extract). Further interpretation of the results was performed based on the comparison of d2 with the control group, as well as between the groups [60].

### 3.9. Statistical Analyses

All data collected in *in vitro* and *in vivo* studies were processed using Microsoft Office Excel for Windows v. 2007 with descriptive, univariate, and test methods in statistics. Data were reported as mean values ± standard deviation (SD). Student *t*-test and one-way ANOVA were used for interpretation of results obtained in *in vivo* experiments. The difference between the compared groups was significant when *p* < 0.05.

## 4. Conclusions

The post-distillation PWE is a rich source of phenols and flavonoids, namely rosmarinic acid. The obtained results from a series of *in vitro* tests indicate that post-distillation residue and other (waste) parts of the plant that are not normally used (e.g., stem, leaf left after distillation) exhibit comparable pharmacological and biochemical (antioxidant, anticholinesterase) effects with peppermint extracts prepared according to the regulations of valid pharmacopeias and monographs. The results of microbiological tests indicate a pronounced bacteriostatic action of all analyzed extracts, which implies the possibility of both standard and PWE application in the food industry. *In vivo* investigation of antioxidant activity showed that the analyzed peppermint extracts exhibit hepatoprotective action, where the most notable results were obtained for the influence on LP and GSH concentration. In pharmacodynamic studies, it was observed that peppermint extracts exhibit a stimulatory effect on the CNS. None of the examined extracts interfered with the metabolism and/or pharmacological effects of midazolam and fluoxetine, which indicates the safety of their concomitant use. Considering the points outlined, PWE can be used as a valuable raw material in the pharmaceutical industry for the isolation of pharmacologically active compounds and the production of various herbal medicinal products, but also in the food industry as a natural preservative.

By endorsing post-distillation waste of aromatic plants, producers could reduce environmental impact and disposal cost while generating additional economic value. The possibility of exploitation of PWE in various industries encourages further optimization of extraction and waste processing. Moreover, further research could be directed towards the *in vivo* efficacy studies and docking studies in the search for the part of the molecules in PWE responsible for the obtained results, especially on the central nervous system.

## Figures and Tables

**Table 1:** The amount of dry extract, total phenolic compounds, and total flavonoid content in analyzed untreated peppermint leaves (P1) and peppermint post-distillation waste extracts (P2–P6) (results are presented as an average value ± standard deviation).

Type of Extract	% Dry Extract (g d.e./100 g of Plant Material)	Total Phenolic Compounds (mg GAE/g d.e.)	Total Flavonoid Content (µg QE/g d.e.)
P1	21.45 ± 0.1	31.65 ± 0.03	39.25 ± 0.002
P2	24.17 ± 0.2	36.68 ± 0.002	31.02 ± 0.003
P3	7.95 ± 0.06	36.75 ± 0.01	48.35 ± 0.004
P4	6.43 ± 0.12	38.73 ± 0.01	67.63 ± 0.007
P5	7.19 ± 0.07	24.14 ± 0.03	10.44 ± 0.003
P6	6.88 ± 0.07	34.29 ± 0.003	19.84 ± 0.006

**Table 2:** Phenolic compounds of peppermint leaves and post-distillation waste extracts.

Type of Extract Concentration (mg/g d.e.)	P1	P2	P3	P4	P5	P6
Gallic acid	0.15	0.09	0.08	0.08	0.11	0.13
Protocatechuic acid	0.27	0.32	0.39	0.46	0.25	0.36
Catechin	0.46	0.32	0.88	0.39	1.41	1.82
Caffeic acid	4.29	1.61	2.09	2.27	2.47	2.53
Chlorogenic acid	0.56	0.66	1.12	1.42	0.82	0.56
Syringic acid	0.07	0.06	0.09	0.12	0.09	0.13
Epicatechin	0.35	0.32	0.47	0.58	0.45	0.63
Ferulic acid	0.24	0.14	0.18	0.22	0.13	0.18
Rutin	0.61	0.45	0.66	0.76	0.5	1.25
Rosmarinic acid	20.51	11.99	18.89	21.19	7.05	10.19
Naringenin	0.18	0.13	0.73	0.72	0.03	0.14
Apigenin	0.51	0.31	3.07	2.98	0.31	0.6

**Table 3:** Antioxidant activity of peppermint post-distillation waste extracts in *in vitro* assays (expressed as IC_50_ in μg/mL for DPPH, OH, and LP tests).

Sample/Assay	DPPH	AAI	OH^•^	LP
P1	2.85	3.11	51.21	3.98
P2	2.25	3.94	65.19	2.49
P3	3.22	2.75	23.25	3.05
P4	2.2	4.02	48.28	1.64
P5	8.45	1.05	66.06	9.19
P6	3.03	2.92	53.61	4.31
BHT	6.95	1.3	/	14.71
Rutin	1.22	7.28	/	/
Rosmarinic acid	0.55	16.16	/	5.73

AAI—Antioxidant activity index; DPPH—2,2-diphenyl-1-picrylhydrazyl; OH^•^—hydroxyl radicals; LP—lipid peroxidation; PG—propyl gallate; BHT—butylated hydroxytoluene.

**Table 4:** Values of biochemical parameters in the liver homogenate of the control group and a group of experimental animals (*n* = 10) treated with selected peppermint extracts (P1, P3, and P4) (results are presented as an average value ± standard deviation).

Parameter	Control		Extract					
			P1		P3		P4	
	Treatment							
	-	CCl_4_	-	CCl_4_	-	CCl_4_	-	CCl_4_
proteins (mg/g of liver homogenate)	318 ± 40.2	397.4 ± 35.4	317.8 ± 27.1	360.4 ± 11.3	317.3 ± 32.0	359.2 ± 14.0	351.2 ± 13.2	392.7 ± 53.2
LP (nmol MDA/mg of proteins)	0.47 ± 0.13	0.98 ± 0.12	0.66 ± 0.07 ^§^	1.26 ± 0.3 ^#^	0.73 ± 0.16	0.97 ± 0.54	0.13 ± 0.03 ^#§^	1.14 ± 0.17
XOD (nmol/mg of proteins min^−1^)	4.71 ± 0.98	11.84 ± 1.45	7.5 ± 1.52 ^§^	11.34 ± 0.72 ^#^	7.63 ± 0.9 ^§^	11.41 ± 2.16 ^#^	7.37 ± 2.49 ^§^	9.54 ± 1.84 ^#^
SOD (IU/mg of proteins) ^a^	0.57 ± 0.11	1.24 ± 0.33 ^#^	0.85 ± 0.29	1.35 ± 0.47	0.86 ± 0.45	1.12 ± 0.29	0.52 ± 0.22 ^§^	0.9 ± 0.42
GSH (nmol/mg of proteins)	1.05 ± 0.31	0.05 ± 0.04 ^#^	1.6 ± 0.53 ^§^	0.29 ± 0.14 ^#^	1.58 ± 0.05 ^§^	0.3 ± 0.08 *^§^	2.75 ± 0.49 ^#§^	0.96 ± 0.28 ^§^
GSH-Px (IU/g of proteins) ^a^	1.93 ± 0.75	3.16 ± 0.86	3.03 ± 1.17	1.41 ± 0.94	2.08 ± 1.58	2.27 ± 1.03	1 ± 0.23 ^§^	2.14 ± 0.88
GSH-R *	12.61 ± 5.58	18.94 ± 0.93	9.49 ± 8.33	19.31 ± 11.86	6.64 ± 2.31 ^§^	18.34 ± 10.41	10.89 ± 12.69	13.52 ± 5.72
GSH-(S)T **	5.48 ± 2.84	16.31 ± 8.87	10.59 ± 6.66	10.12 ± 5.44	3.34 ± 1.16	14.43 ± 10.63	5.49 ± 1.89	7.39 ± 3.64

P1—standard extract; P3—deodorized leaf extract (45% ethanol); P4—deodorized leaf extract (75% ethanol); LP—lipid peroxidation; XOD—xanthine oxidase; SOD—superoxide dismutase; GSH—gluthatione; GSH-Px—gluthatione peroxidase; * GSH-R—gluthatione reductase (results were expressed in nmol NADPH/mg of proteins min^−1^); GSH-(S)T—** gluthatione-S-transpherase (results were expressed in nmol conjugate/mg of proteins min^−1^); MDA—malondialdehyde; ^a^ IU—international units; ^#^—*t*-test confirmed statistically significant difference compared to the control group (*p* < 0.01); ^§^—*t*-test confirmed statistically significant difference compared to the control CCl_4_ (*p* < 0.01).

**Table 5:** Minimal inhibitory concentrations (mg TP/mL) of investigated peppermint extracts on selected bacterial strains.

	*Staphylococcus aureus*	*Salmonella* Infantis	*Escherichia coli*	*Bacillus cereus*
P1	0.12	0.125	0.26	0.35
P2	0.15	0.15	0.3	0.59
P3	0.07	0.15	0.18	0.07
P4	0.15	0.21	0.15	0.11
P5	0.13	0.39	0.20	0.27
P6	0.28	0.55	0.19	0.28
BHT (mg/mL)	0.55	0.55	0.55	0.28
Rosmarinic acid (mg/mL)	5	2.5	2.5	5

**Table 6:** Minimal inhibitory concentrations (mg TP/mL) of selected peppermint extracts, rosmarinic acid (mg/mL), and selected antibiotics (µg/mL) on *Campylobacter jejuni*.

	*Campylobacter jejuni*
P1	0.13
P3	0.07
Rosmarinic acid	1.25
Ciprofloxacin	0.25
Erythromycin	0.25

**Table 7:** Retention time (s) as a measure of midazolam effect on motor coordination in controls and peppermint extract-pretreated groups of animals (*n* = 10) (results are presented as an average value ± standard deviation).

Extract	Timeframe After Midazolam Application (min)				
	1–4	5–8	10–13	15–18	20–23
P1-control	74.78 ± 79.31	134.5 ± 69.59	180.0 ± 0.0	180.0 ± 0.0	180.0 ± 0.0
P1-drug	88.5 ± 81.4 ^#^	115.33 ± 75.3	132.8 ± 74.1	149.7 ± 60.46	170.7 ± 27.26
P1-extract	142.2 ± 61.03	169.8 ± 30.67	174.4 ± 16.67	180.0 ± 0.0	180.0 ± 0.0
P1-interaction	146.7 ± 67.5	152.55 ± 59.2	166.67 ± 40.0	170.9 ± 23.33	180.0 ± 0.0
P3-control	79.6 ± 75.8	134 ± 74.32	159.2 ± 45.53	180.0 ± 0.0	180.0 ± 0.0
P3-drug	80.7 ± 75.4 ^#^	112.6 ± 86.75	140.3 ± 69.06	164.6 ± 48.7	180.0 ± 0.0
P3-extract	180.0 ± 0.0	180.0 ± 0.0	180.0 ± 0.0	180.0 ± 0.0	180.0 ± 0.0
P3-interaction	118.8 ± 73.13	146.3 ± 71.11	178.5 ± 4.74	180.0 ± 0.0	180.0 ± 0.0
P4-control	97.9 ± 76.4	148 ± 67.48	164.3 ± 49.65	168.3 ± 37.0	180.0 ± 0.0
P4-drug	180.0 ± 0.0 ^#^	180.0 ± 0.0 ^#^	180.0 ± 0.0	180.0 ± 0.0	180.0 ± 0.0
P4-extract	83.7 ± 77.49	95.4 ± 87.37	151.6 ± 48.02	169.2 ± 34.15	170.6 ± 29.73
P4-interaction	112.9 ± 75.18	127.6 ± 84.39	141.1 ± 67.72	177.7 ± 7.27	180.0 ± 0.0

^#^—*t*-test confirmed the statistically significant difference when compared to the control (*p* < 0.05).

**Table 8:** Immobilization time (s) in the Tail Suspension Test, measured after the administration of the drug (fluoxetine) and investigated peppermint extracts (results are presented as an average value ± standard deviation).

Type of Treatment	Immobilization Time (s)
P1—control	118.33 ± 36.6
P1—drug	51.25 ± 31.9 ^#^
P1—extract	101.25 ± 32.59
P1—drug + extract	62.12 ± 42.8 ^#^
P3—control	112.8 ± 51.8
P3—drug	69.8 ± 49.3 ^#^
P3—extract	109 ± 50.3
P3—drug + extract	74.1 ± 53.0 ^#^
P4—control	105.2 ± 46.15
P4—drug	59.6 ± 50.9 ^#^
P4—extract	97.1 ± 52.7
P4—drug + extract	56 ± 53.3 ^#^

^#^—*t*-test confirmed statistically significant difference (*p* < 0.05) when compared to the control group.

**Table 9:** Elevated plus maze (EPM) test parameters recorded in the control and groups treated with the drug (diazepam) and peppermint extracts (results are presented as an average value ± standard deviation).

Extracts	EPM Parameters				
	nO	nE	tO (s)	tE (s)	tCP (s)
Control	0.57 ± 0.79	9.57 ± 1.81	2.71 ± 3.64	154.71 ± 26.46	142.57 ± 26.02
Drug	6.43 ± 6.97 ^#^	12.14 ± 3.39	38.43 ± 50.73 ^#^	128.57 ± 50.04	118.71 ± 35.96
P1-extract	1.57 ± 1.62	10.14 ± 1.86	6.14 ± 6.41	164.43 ± 41.66	129.43 ± 44.97
P3-extract	3 ± 2.08 ^#^	8 ± 2.24 ^§^	14.57 ± 14.42 ^#^	144.86 ± 35.82	140.57 ± 27.96
P4-extract	2 ± 1.73 ^#^	8.71 ± 3.15 ^§^	6.14 ± 8.23 ^§^	142.86 ± 43.33	151 ± 44.86

nO—number of open arm entries; nE—number of enclosed arm entries; tO—time spent in open arms; tE—time spent in enclosed arms; tCP—time spent on central platform. ^#^—*t*-test confirmed the statistically significant difference (*p* < 0.05) compared to the control. ^§^—*t*-test confirmed the statistically significant difference (*p* < 0.05) compared to the drug (diazepam).

**Table 10:** Extraction procedure and abbreviations for examined peppermint extracts.

Abbreviations	Extract Preparation
P1	maceration of the leaves in 45% ethanol for 24 h—officially prepared (standard) extract
P2	decoction (water extract) of the remaining after hydro-distillation of peppermint leaves
P3	maceration of deodorized peppermint leaves (essential oil was removed with hydro-distillation) in 45% ethanol for 24 h
P4	maceration of deodorized peppermint leaves (essential oil was removed with hydro-distillation) in 75% ethanol for 24 h
P5	maceration of ground peppermint stems in 45% ethanol for 24 h
P6	maceration of ground peppermint stems in 75% ethanol for 24 h

**Table 11:** Strains of microorganisms/bacterial strains used in determining the antimicrobial activity of the tested extracts.

Bacterial Strain	Source
*Staphylococcus aureus*	ATCC 25923
*Bacillus cereus*	ŽMJ 164
*Salmonella* Infantis	ŽMJ 106
*Escherichia coli*	ŽMJ 370
*Campylobacter jejuni*	NCTC 11168

ATCC—American Type of Culture Collection; ŽMJ—strain from Biotechnical Faculty, University of Ljubljana, Slovenia; and NCTC—National Collection of Type Cultures.

## Data Availability

Data is contained within the article.
